# Effects of Irritation on Motor Region of the Brain

**Published:** 1882-09

**Authors:** 


### ARTICLE VI.

### *Effects of Irritation on the Motor Region of the Brain.*

The present generation of physicians have been taught to believe that owing to a decussation of fibers at the base of the brain, each hemisphere holds its relation to the opposite side of the body and not on its own side. But we are now required to modify our views on this subject, if not to throw them aside. According to the London *Lancet*, Dr. Brown-Sequard repudiates the old theory. The numerous researches which he has undertaken during the last four years seem to him to involve conclusions exactly contrary to the opinions which are universally received, For example, against the assertion that the irritation of the motor region of the brain uniformly produces movements in the limbs on the opposite side, he opposes certain experiments of his own. These show that irritation of one side of the pons Varolii or of the medulla, even of the anterior pyramid, causes eight or nine times out of ten movements of the limbs on the same side, and the same effect is observed when, after a transverse division of one-half of the medulla, the superior part of the pons is stimulated, mechanically or by electricity, in the part considered as motor. Irritation of the cerebral peduncle in the part considered as motor often causes movements of the limbs on the same side. This result occurs five or six times in ten when the stimulation is applied to the upper part. If the fibers are galvanized which pass from the corona radiata or corpus striatum to the peduncle, movements are often observed on the corresponding side of the body. If these parts are divided transversely on the right side or on the left, the mechanical excitation thus produced rarely causes movement, but when it does the effect is usually manifested on the same side as the irritation. Even stimulation of the motor zone of the cortez, as Couty has shown, sometimes causes movements on the corresponding side. Moreover, Dr. Brown-Sequard has repeatedly showd that if this zone is galvanized, after the lateral half of the medulla or of the pons Varolii is divided, the movements in the opposite limbs, instead of being prevented by this section, occur with still greater force than before the division of those conductors which have been believed to be alone capable of transmitting the stimulation of this zone to the limbs.

According to received doctrines, if one lateral half of the cervical cord is divided at the level of the second pair of nerves, and different parts of the brain are then stimulated, mechanically or electrically, on the same or on the opposite side to the spinal lesion, no movement should occur, or only a very slight movement in the members on the same side as the lesion. But Dr. Brown-Sequard finds that, under these circumstances, stimulation of the brain causes energetic movements of the limbs, such as "bipedal" movement, diagonal or lateral, to the right or left, or a movement of three, or even of four limbs. He concludes from this that one-half of the cord will suffice to transmit to the limbs, on both sides of the body, the excitation caused by stimulation of the opposite half of the brain.

According to received doctrines the transverse section of the two lateral halves of the base of the brain; the one section at a distance of one centimetre above or below the other, ought to destroy all or almost all communication between the spinal cord and the portions of the brain above the higher section, so that mechanical or chemical excitation of the cortex should cause no effect on the limbs. But Dr. Brown-Sequard asserts that under these circumstances not only does stimulation of the motor centres act energetically upon the limbs, but the same effect is produced by stimulation of the parts which are not considered to be motor, such as the optico-striate bodies. In this case, also, the effect is usually most marked on the same side as that stimulated. An analysis which Dr. Brown-Sequard has made of 500 cases of unilateral convulsions in consequence of varied lesions of the brain, show that the same result is true of man as of animals. Irritation of the base of the brain and the adjacent motor regions causes convulsions more frequently on the side irritated than on the other. The superficial parts of the brain, it is true, produce chiefly crossed convulsions, but irritation in all parts *may* cause convulsion on the same side.

The conclusions drawn by Dr. Brown-Sequard are that one of the chief foundations for the theory of psycho-motor centers, and of the crossed functional relation between the hemispheres and the limbs, must be considered to have lost its value; and, secondly, that the excito-moto-zone of the cerebral surface, and indeed all the excitable parts of the brain, are capable of putting in action the limbs of the same side, as well as those of the opposite side, and that they may produce these effects after the transverse division of one-half of the pons Varolii, of the medulla, or of the cervical cord, and even after two sections of the base, one of the right half and the other of the left, provided a certain interval exists between the two.—*Pacific Med. and Surg. Journal.*

